# ^13^C Isotope Labelling to Follow the Flux of Photorespiratory Intermediates

**DOI:** 10.3390/plants10030427

**Published:** 2021-02-24

**Authors:** Cyril Abadie, Guillaume Tcherkez

**Affiliations:** 1 Institut de Recherche en Horticulture et Semences, Université d’Angers, INRAe, 42 rue Georges Morel, 49070 Beaucouzé, France; cyril.abadie@inrae.fr; 2 Research School of Biology, College of Science, Australian National University, Canberra 2601, Australia

**Keywords:** photorespiration, photosynthesis, isotope, labelling, nuclear magnetic resonance, high resolution mass spectrometry

## Abstract

Measuring the carbon flux through metabolic pathways in intact illuminated leaves remains challenging because of, e.g., isotopic dilution by endogenous metabolites, the impossibility to reach isotopic steady state, and the occurrence of multiple pools. In the case of photorespiratory intermediates, our knowledge of the partitioning between photorespiratory recycling, storage, and utilization by other pathways is thus rather limited. There has been some controversy as to whether photorespiratory glycine and serine may not be recycled, thus changing the apparent stoichiometric coefficient between photorespiratory O_2_ fixation and CO_2_ release. We describe here an isotopic method to trace the fates of glycine, serine and glycerate, taking advantage of positional ^13^C content with NMR and isotopic analyses by LC–MS. This technique is well-adapted to show that the proportion of glycerate, serine and glycine molecules escaping photorespiratory recycling is very small.

## 1. Introduction

Illuminated leaves assimilate CO_2_ via gross photosynthesis (carboxylation) but also release CO_2_ via photorespiration and day respiration. Experimental methods to determine the rate of photorespiration are not straightforward [1]. The photorespiration rate (Φ) is often inferred from the CO_2_ mole fraction at the carboxylation sites (*c_c_*), allowing calculation of the carboxylation rate (*v_c_*). In fact, assuming that two oxygenation events are required for each photorespiratory CO_2_ release, we have the general relationships [2]:

(1) $$\Phi =\frac{v_{o}}{2}\;\mathrm{where}\;v_{o}=\frac{2v_{c}\Gamma *}{c_{c}}\;\mathrm{and}\;v_{c}=\frac{A+R_{d}}{1-\frac{\Gamma *}{c_{c}}}$$

In (1), Γ* is the CO_2_ compensation point in the absence of day respiration, *A* is net assimilation, and *R_d_* is day respiration. It is apparent that the stoichiometric coefficient between oxygen fixation (*v_o_*) and Φ is 2. If the stoichiometric coefficient differs substantially from 2, estimates of both *v_o_* and Φ are affected. Enzymatic biochemistry of photorespiratory reactions requires two molecules of glycine to synthesize one molecule of CO_2_ [3] and therefore, it is generally assumed that a stoichiometric coefficient of 2 is applicable. In recent years, there has been some controversy as to whether some glycine molecules produced by photorespiration are not converted to serine and thus, the apparent stoichiometric coefficient is actually not equal to 2 (for a specific discussion, see [4]). Solid-state NMR analyses have suggested that a substantial proportion of glycine is used by other pathways, typically protein synthesis [5]. Similarly, it has been suggested by modelling that serine, in addition to glycine, can be used by other pathways to a substantial extent, and this loss of N atoms has to be compensated for by extra electrons [6]. Radioactive labelling (with ^14^CO_2_) has shown incomplete turn-over in serine, suggesting the existence of several pools [7]. This highlights the need to find methods to precisely assess the fate of photorespiratory intermediates and determine if they can effectively escape from photorespiratory recycling. Recently, we used an ^15^N-NMR based method to show that in sunflower, the stoichiometric coefficient between Φ and *v_o_* is always very close to two, regardless of the CO_2_ and O_2_ mole fractions applied during gas exchange [8]. It should be recognized that although useful, this technique is not applicable on a routine basis due to both the technicity and acquisition time of ^15^N-NMR analysis. Other techniques can be used to monitor photorespiratory fluxes, such as ^18^O labelling of leaf H_2_O or atmospheric O_2_ [9,10,11,12]. These techniques have two major problems: first, ^18^O isotopic products are expensive; second, they can provide access to *v_o_* but not reliably to photorespiratory intermediates due to isotopic exchange between water and –OH and –COOH groups in metabolites.

**Figure 1:**
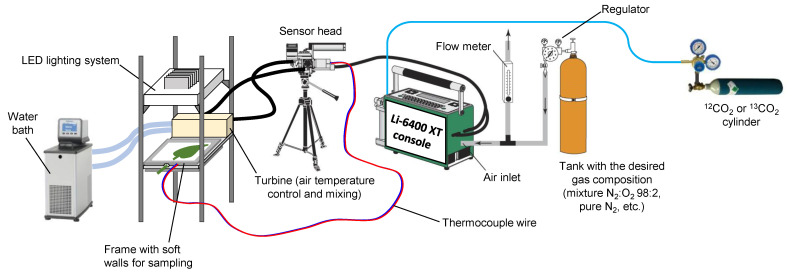
Experimental setup to perform isotopic labelling and prepare leaf samples that have been instant-frozen with liquid N_2_ spraying (thanks to the breakable soft and transparent walls of the leaf chamber). The system includes a portable gas exchange system allowing the control of CO_2_ and H_2_O mole fractions. The chamber can accommodate relatively large leaves and thus allows preparation of samples of sufficient size for NMR analysis. Redrawn from [17].

**Figure 2:**
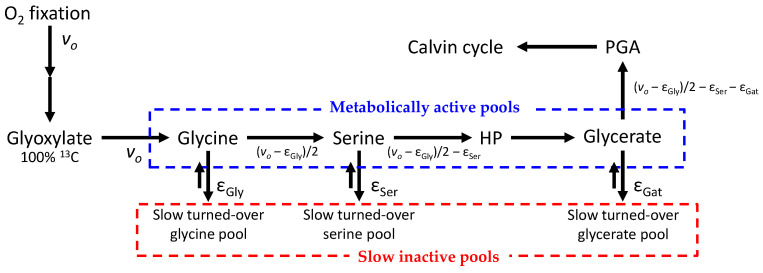
Simplified metabolic pathway used in calculations. In this paper, the objective is to estimate the flux of molecules escaping the photorespiratory recycling—that is, ε values (ε_Gly_, ε_Ser_ and ε_Gat_). The oxygenation rate is denoted as *v_o_*. Glycine, serine and glycerate are subdivided into two pools—one metabolically active pool of constant size, and a slow “inactive” pool of increasing size (due to build-up). Since the leaf pool of glyoxylate is very small (undetectable by ^13^C NMR), it is assumed that it does not accumulate and is turned over rapidly so that its ^13^C enrichment is 100%. HP, hydroxypyruvate; PGA, glycerate 3-phosphate.

Therefore, it is desirable to find alternative, more approachable methods to trace photorespiratory intermediates so as to calculate metabolic fluxes. ^13^C-labelling with ^13^CO_2_ has been performed before to infer general flux patterns in *Arabidopsis* rosettes but build-up rates from photorespiration have not been directly quantified [13,14,15]. Here, we propose a method based on ^13^C, which is easily implementable using routine ^13^C-NMR and LC–MS analyses. This method has been used in sunflower leaves [16] and in the Materials and Methods of the paper presented here, we provide a more precise description and discuss pros and cons of the techniques used. The experimental labelling system is illustrated in Figure 1, and the principle of ^13^C-based calculations is shown in Figure 2 (further details are provided in the Materials and Methods section below, Section 4). This isotopic method allows estimation of the rate associated with the accumulation of serine, glycerate and glycine in leaves, three metabolites that are relatively abundant in sunflower leaves. It demonstrates that under several gaseous conditions, carbon escaping from the photorespiratory cycle represents a very small flux compared to *v_o_*.

## 2. Results

Our method has been applied to sunflower leaves, using ^13^CO_2_ (99% ^13^C) under a range of atmosphere compositions (CO_2_/O_2_ mole fraction ratios) to vary the photorespiration rate (Table 1)—from very high photorespiration (in 100% O_2_ as a background gas) to negligible photorespiratory flux (in 100% N_2_ as a background gas). This section shows typical results obtained therefrom, including original data (^13^C signals in NMR) and the output of calculations—i.e., the flux of molecules escaping from photorespiratory recycling (denoted as ε in Figure 1).

### 2.1. Isotopic Signals in Photorespiratory Intermediates

**Figure 3:**
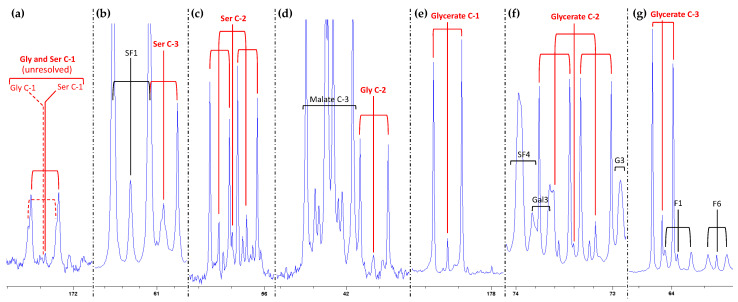
^13^C NMR signals of C-atom positions in glycine (**a**,**d**), serine (**a**–**c**) and glycerate (**e**–**g**) in sunflower leaves, under standard conditions (21% O_2_, 380 µmol mol^−1^ CO_2_). Spectra were acquired in the presence of EDTA to allow resolution of –COOH groups. Note, however, the nearly complete overlapping of glycine and serine C-1 signals (**a**). The decomposition of signals caused by ^13^C–^13^C interactions is shown in red. Overlapping with other compounds is shown in black. The left peak of serine C-3 (**b**) overlaps with one peak of the fructosyl moiety of sucrose, and glycerate C-3 (**g**) partly overlaps with the C-1 atom of fructose. Abbreviations: F1, fructose C-1; F6, fructose C-6; G3, glucose C-3; Gal3, galactose C-3; SF1, fructosyl moiety of sucrose C-1; SF4, fructosyl moiety of sucrose C-4. Different magnifications were used to facilitate reading.

NMR signals of glycine, serine and glycerate are easily visible using standard ^13^C-NMR analysis, and they are shown in Figure 3. Because the ^13^C-labelling was performed with ^13^CO_2_, Calvin cycle intermediates (including ribulose 1,5-bisphosphate, RuBP) were rapidly ^13^C-labelled in all C-atom positions, meaning that photosynthetic and photorespiratory products ended up being ^13^C-labelled at several positions within the same molecule. This led to multiplets in NMR signals due to spin–spin interactions between neighbor ^13^C atoms. In principle, single and double spin–spin interactions form doublets and quadruplets, respectively. However, when the spin–spin coupling constant between neighboring C-atoms is similar, overlapping may lead to a simplification of quadruplets into triplets (for a detailed explanation of NMR ^13^C-signal multiplicity, see [18]). Of course, for each C-atom position, a metabolite pool contains a mixture of molecular populations: mono- (^13^C_1_; no labelled neighbor C-atom), bi- (^13^C_2_; one neighbor ^13^C) and trilabelled (^13^C_3_; two neighbor ^13^C). The final signal found by NMR is a multiplet with up to 11 peaks depending on ^13^C–^13^C coupling constants (typically if there is strong dissymmetry).

In the case of photorespiratory metabolites, there were quite important differences between C-atom positions. First, glycine and serine –COOH groups formed a common peak, a broadened triplet at a chemical shift (δ) of about 173 ppm (Figure 3a). In that triplet, the two high side peaks represent –^13^COOH groups coupled to a neighbor ^13^C (labelled α carbon) while the small central peak represents –^13^COOH groups with a nonlabelled neighbor (^12^C). The fact that the central peak is small shows that a substantial proportion of glycine and serine molecules have multiple ^13^C atoms. The C-3 atom of serine (Figure 3b) also generated a triplet (two side peaks and a small central peak), one of the side peaks being overlapped with a peak of sucrose.

The C-2 atoms in both glycine and serine (Figure 3c,d) were well-resolved and could be distinguished from other metabolites. For glycerate, C-2 and C-3 atoms were close to sugars (sucrose, glucose, galactose or fructose) but could be resolved, with a 7-uplet (C-2) and a triplet (C-3) (Figure 3f,g). As with glycine and serine, such a peak multiplicity shows that the glycerate pool was a mixture of isotopologues (i.e., molecular forms with different C-atom labelling patterns: mono-, bi- or trilabelled).

### 2.2. Isotopologue Distribution

**Figure 4:**
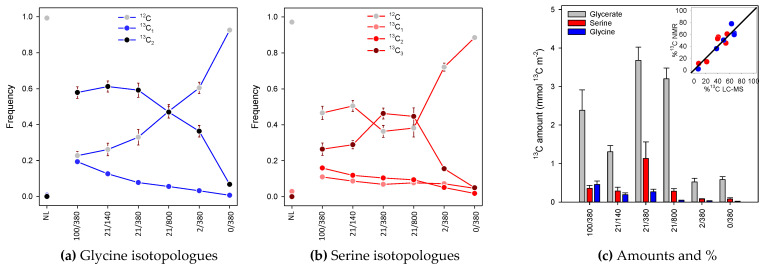
^13^C analysis of serine, glycine and glycerate: ^13^C-species of glycine (**a**) and serine (**b**) analyzed by LC–MS and ^13^C amount measured by NMR (in mmol ^13^C m^−2^) along with the ^13^C percentage (inset). Isotopologue abundance is shown in frequency i.e., mole fraction of total (**a**,**b**). The inset in (**c**) compares NMR and LC–MS-derived values. The solid black line stands for the 1:1 line. In (**c**), the amount shown accounts for the number of C-atoms in the molecule. NL, no labelling (experiment at natural abundance).

The isotopologue distribution can be resolved from NMR signals by calculation (i.e., computing the relative quantity of mono-, bi- or trilabelled forms from peak decomposition). However, this can be measured directly using routine analysis by exact mass LC–MS, which gives signals that are mono-, bi- or trilabelled (+1.003355 *m/z* unit per ^13^C). LC–MS in positive mode gives access to serine and glycine analysis (while glycerate would require analysis in negative mode), and results are shown in Figure 4. As expected, there was a general decline in ^12^C (nonlabelled) glycine and serine isotopologue abundance as photorespiration increased (from right to left in Figure 4). However, the most labelled isotopologue, ^13^C_3_-serine, did not increase progressively but peaked under standard conditions (21% O_2_, 380 µmol mol^−1^) and high CO_2_ (21% O_2_, 800 µmol mol^−1^ CO_2_). This simply shows that maximal labelling in serine C-atoms was observed when the ^13^C flux through photorespiratory intermediates was maximal—that is, when the product of ^13^C abundance in RuBP and oxygenation rate (*v_o_*) was optimal. When photorespiration is very high (e.g., under 100% O_2_), *v_o_* is at the highest but photosynthesis is low and so is the ^13^C input. When photorespiration is low (e.g., 0% O_2_), the ^13^C input is high but *v_o_* is far too small to turn-over photorespiratory intermediates. This effect was visible in serine (Figure 4b) but not in glycine (Figure 4a) because the pool size of serine is larger than that of glycine is sunflower leaves [8]. For the same reason, even though photorespiration was maximal under 100% O_2_, the ^13^C_2_-glycine isotopologue kept the same proportion as under 21% O_2_ 140 µmol mol^−1^ CO_2_.

While LC–MS analyses can resolve isotopologue proportions, they are not ideal to quantify ^13^C amounts (in moles) precisely. In fact, absolute quantitation with LC–MS works best with calibration curves applied to internal standards and, typically, isotopically substituted standards are necessary. Here, we avoided this method not to complicate isotopic patterns. Furthermore, NMR provides a direct measurement of ^13^C content since peak integrals are proportional to the number of ^13^C nuclei at each position. ^13^C amounts represented by serine, glycine and glycerate are shown in Figure 4c. In most cases, the ^13^C amount was very small, of less than 1.5 mmol m^−2^. Glycerate ^13^C content peaked under standard, high O_2_ and high CO_2_ conditions. The fact that the amount of ^13^C represented by glycerate was not very high at low CO_2_ (21% O_2_, 140 µmol mol^−1^ CO_2_) came from the fact that at low CO_2_, the ^13^C input is rather small (a similar effect is described above for the ^13^C_3_ isotopologue of serine). We also assessed consistency by comparing the %^13^C obtained by LC–MS and NMR and found that points were very close to the 1:1 line (Figure 4c, inset). 

### 2.3. Metabolic Fluxes

**Figure 5:**
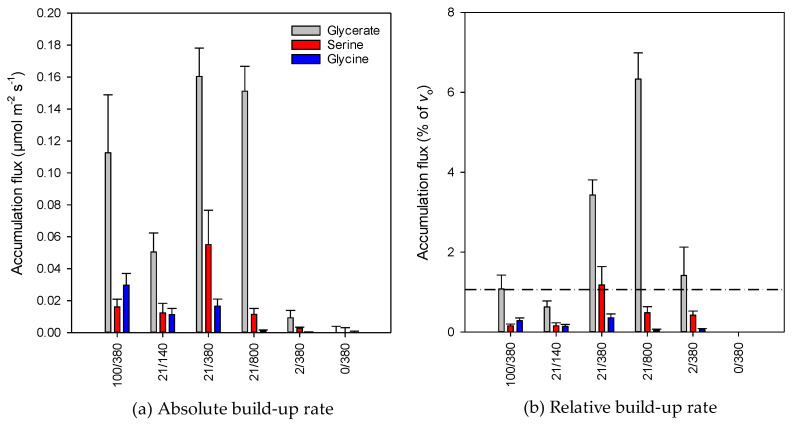
Accumulation flux (build-up rate) of photorespiratory intermediates (denoted as ε in Figure 2): absolute value in µmol m^−2^ s^−1^ (**a**) and flux relative to oxygenation rate (**b**). In (**b**), the dash-dotted horizontal line stands for a fixed proportion of 1%.

Isotopic data (percentage, amount) were then used to compute build-up rates associated with glycine, serine and glycerate (Figure 5a). Taken as a whole, there was an increase in the build-up rate as photorespiration increased, from hardly detectable build-up under 0% O_2_ to ≥ 0.10 µmol m^−2^ s^−1^ at normal and high photorespiration. It is worth noting that the maximal build-up rate of serine and glycerate was found under standard conditions, suggesting that at high photorespiration (high oxygen or low CO_2_), glycine conversion to serine was less efficient and/or serine and glycerate recycling was more efficient. In particular, glycine accumulation was higher under 100% O_2_, suggesting that glycine conversion to serine by glycine decarboxylase/serine hydroxymethyltransferase (GDC-SHMT) was less efficient at high photorespiration. However, when build-up rates were expressed in percentage of *v_o_* (which represents glycine production rate from glyoxylate), values were always lower than 1%, except for glycerate under standard conditions and low CO_2_ (Figure 5b). Glycine accumulation was always less than 0.5% of *v_o_*.

## 3. Discussion

### 3.1. Pros and Cons of the ^13^C-Based Isotopic Method

The method presented here to determine serine, glycine and glycerate build-up rates in illuminated leaves has several advantages. First, using NMR provides a direct measurement of ^13^C content at each C-atom position. Once a proper internal standard (here, maleate) and a response curve (at different chemical shifts) have been verified, NMR signals can be easily converted into moles of ^13^C. The use of EDTA in samples further allows the resolution of –COOH groups, as we demonstrated before [19]. Second, sample analysis by NMR does not require lengthy preparation (the neutralized perchloric acid extract can be analyzed directly after centrifugation and resuspension in NMR tube). Additionally, it is not destructive and thus makes extra analysis possible, e.g., with LC–MS, to assess isotopologue distribution and % ^13^C values (a similar process has been used for ^33^S isotopic analysis by both NMR and LC–MS, [20]). Of course, since our method uses ^13^C-labelling, it is necessary to purchase ^13^CO_2_, which is rather expensive, but this issue is inherent to all metabolic flux analyses. 

There are, however, some potential disadvantages. First, NMR analyses require large samples (1–3 g fresh weight, i.e., 50–120 cm^2^) and a rather long time. In fact, our NMR sequence (inverse gated pulse program with D1 relaxation delay of more than 1 s) and thus each sample analysis took about 12 h. Second, there can be some overlapping in NMR signals. This is typically the case for serine and glycerate (Figure 3). Nevertheless, the contribution of other metabolites can be usually sorted out taking advantage of signal symmetry (one of the side peaks can still be integrated while the other one is not exploitable because of overlapping) [18]. Third, analogous metabolites can have very close chemical shifts, making the use of signals more difficult. Here, this is potentially the case for glycerate, the chemical shifts of which are close to that of glycerate 3-phosphate (PGA, the product of carboxylation). In C-3, the presence of the phosphate group in PGA leads to a signal downfield (higher chemical shift) at about 67 ppm (while glycerate C-3 is at 64 ppm). However, C-1 and C-2 atoms have similar chemical shifts (179 and 73 ppm, respectively). Although the contribution of PGA is probably modest in our case (sunflower leaves do not have large amounts of PGA, within 25–600 µmol m^−2^ [7,21] while glycerate content is about 5 mmol m^−2^), ^13^C signals in C-1 and C-2 probably integrate some PGA in addition to glycerate. As such, our glycerate build-up rates must be seen as maximal possible values. Further analyses with LC–MS in negative mode and ionic LC could be proposed as an alternative method to analyze glycerate and assess isotopic differences with PGA. 

In terms of calculations, our method is associated with several assumptions (see Materials and Methods for details). However, they do not introduce significant numerical errors. First, the oxygenation rate *v_o_* was estimated using the intercellular CO_2_ mole fraction and the *c_i_*-based compensation point in the absence of day respiration. Any imprecision on *v_o_* only has a small impact on the calculated build-up rate since the mathematical term that contains *v_o_* is numerically small (<10 except in 0% O_2_; in 0% O_2_, *v_o_* is tiny and there is a very small ^13^C accumulation as seen by NMR) compared to the other terms involved in calculations. Therefore, the fact that *v_o_* was not obtained through a proper estimate of *c_c_* was not problematic here. Second, the active glycine pool size was estimated using the ^13^C amount seen under 2% (corrected for natural abundance) since under these conditions, there is no glycine build-up [8,16] (it is effectively negligible, Figure 5a). The inactive glycine pool size at time *t* = 0 (i.e., at the start of gas exchange experiment, thus just before glycine build-up started) was estimated using the observed ^13^C amount (natural abundance) in 0% O_2_ since it was assumed that no significant glycine flux occurred in this condition. We believe that this assumption is reasonable, as demonstrated by ^15^N labelling [8,22].

### 3.2. Possible Consequences of Accumulation of Photorespiratory Intermediates

Our method allows the determination of the build-up rates of glycine and serine and thus to monitor the status of the glycine-to-serine conversion in photorespiration. The ^13^C-analysis using NMR is sensitive enough to measure small build-up rates—here, typically in the order of, or lower than 0.1 µmol m^−2^ s^−1^. This value is small and shows that even at very high photorespiration, glycine and serine metabolism is very efficient. It thus suggests that the build-up of photorespiratory intermediates is too small to change significantly the stoichiometric coefficient of 2 used in calculations (Equation (1)). This agrees with the direct measurement of this coefficient using in vivo ^15^N tracing [8]. Using modelling, it has been proposed that up to 40% of serine or glycine can escape photorespiratory recycling, with huge consequences on nitrogen assimilation and the stoichiometric coefficient of Equation (1) [6]. While it is true that serine and glycine accumulation traps N atoms and thus implies extra N assimilation to keep glutamate homeostasis, it is unlikely that the N imbalance is so high. When expressed relative to *v_o_*, all ε values are ≤1% for both glycine and serine (Figure 5). 

We also recognize that, here, NMR analysis of perchloric extracts only gives access to soluble metabolites—that is, free serine and free glycine. It is possible that some serine and glycine molecules were used to synthesize proteins and thus build-up rates ε calculated here could have been underestimated. Using solid-state NMR, it has been suggested that protein synthesis consumes a substantial proportion of photorespiratory glycine [5]. However, this effect is rather unlikely. Precise measurements using ^13^C labelling have shown that protein synthesis is within 0.05–0.2 µmol m^−2^ s^−1^ in *Arabidopsis* rosettes [23]. Of course, this flux is likely to vary with the developmental stage and growth rate [24]. Still, since glycine and serine represent ≈7% of amino acid residues in proteins, the flux represented by protein synthesis is unlikely to exceed 0.015 µmol m^−2^ s^−1^. This value indicates that the consumption of glycine and serine by protein synthesis is almost negligible.

## 4. Materials and Methods

### 4.1. Gas Exchange System

The gas exchange system is schematized in Figure 1. Gas exchange experiment with labelling was carried out under controlled CO_2_/O_2_ conditions using a chamber coupled to the portable photosynthetic system Li-6400-XT (Licor Biosciences). This chamber had soft and transparent walls to allow facile instant sampling by liquid nitrogen spraying as described previously [25]. Carbon dioxide was provided to the Li-6400-XT using a CO_2_ cylinder regulated at 15 bars, either from Boc Edwards (ordinary CO_2_) or Sigma Aldrich (CO_2_ at 99% ^13^C). The atmosphere in the chamber was at 80% relative humidity and 21–23 °C air temperature, with incident light (photosynthetically active radiation, PAR) of 400 µmol m^−2^ s^−1^. The duration of isotopic labelling was 2 h after having reached steady photosynthesis using ordinary CO_2_. Gaseous conditions used in the present study are summarized in Table 1.

### 4.2. NMR Analysis

Frozen leaf samples were extracted and analyzed as in [17]. Briefly, perchloric acid extracts were prepared in liquid nitrogen with maleate (125 µmol per sample, internal standard). After centrifugation, the pellet was re-extracted with perchloric acid and centrifuged. The two supernatants were combined, pH was adjusted to 5 with potassium bicarbonate and frozen-dried. Then, the sample was resuspended in EDTA (15 mM), pH was adjusted to 7 with KOH and centrifuged. Subsequently, 550 µL of supernatant was collected, 50 µL D_2_O was added and the sample was poured in the NMR tube. Samples were analyzed with an NMR spectrometer Advance 700 Mz (Bruker Biospin). NMR analyses were performed at 25 °C without tube spinning, using proton-decoupled (decoupling sequence waltz16) carbon pulse program (zgig) with 90° pulses for ^13^C of 10 µs at 50 W, 0.9 s acquisition time, 65 k size of FID, and a relaxation delay (D1) of 1.2 s, 20,000 scans. In case the response of individual peaks at different chemical shifts was not perfectly identical, signals were corrected using standards at known concentrations of standards. NMR data presented in this paper are mean ± SD of *n* = 3 replicates.

### 4.3. LC–MS Analyses

LC–MS analyses were carried out as in [20]. Briefly, liquid chromatography was performed using a ZIC^®^-HILIC column with a column guard at 30 °C (oven temperature) in the LC system UHPLC^+^ Ultimate 3000 (Dionex-Thermo Scientific). Aliquots from extracts used for NMR were diluted 10 times in water/acetonitrile and trifluoromethyl phenylalanine was added as the internal standard. Samples were kept at 4 °C (sample tray temperature). Then, 1 µL was injected and elution was carried out at a flow rate of 0.3 mL min^−1^ with a binary gradient made of acetonitrile and water (eluent A was 25:75 v:v and eluent B was 95:5 v:v) with ammonium acetate (5 mM). Mass spec analyses were carried out with the Orbitrap Q Exactive Plus (Thermo Fisher Scientific) with a HESI-II probe operated in positive polarity using the full MS scan mode (source voltage: 3500 V, resolution: 70,000, AGC target: 1 × 10^6^, mass scan range: 60–600 m/z, sheath gas: 40, auxiliary gas: 10, sweep gas: 1.5, probe temperature: 300 °C, capillary temperature: 250 °C and S-lens RF level: 50). Mass calibration was performed with the LTQ-ESI positive ion calibration solution (Pierce^®^, Thermo Fisher Scientific) immediately before each analysis batch. The software Xcalibur was used to handle LC–MS data. LC–MS data presented in the paper are mean ± SD of *n* = 6 replicates.

Note that specific precautions have to be taken for LC–MS analyses. First, the eluent in the LC column must not contain pure acetonitrile at any time, a small percentage of water being necessary even for washing the system between sample batches. Second, since the eluent contains ammonium acetate which has some propensity to crystallize, column pressure must be monitored regularly, and it is desirable to have a flow through the LC column permanently. Third, it is important to ensure the pH of samples is properly adjusted to 7 (as mentioned above) before dilution in water:acetonitrile. In fact, even slight variations in pH may impact on ionization and thus on MS analysis. Note that here, we report data obtained in full MS (no fragmentation). A separate injection with fragmentation (AIF) is possible, allowing access to ^13^C content in molecular fragments. In the case of small molecules (glycine and serine), fragmentation is not feasible (glycine) or accessible by NMR (serine). Further information on exploiting fragmentation to access intramolecular ^13^C contents is provided in [17,20]. 

### 4.4. Calculations

The percentage in ^13^C was calculated from NMR data using two methods: (i) using the ratio of NMR signals—if the signal at the C-atom position obtained upon ^13^C-labelling is denoted as ^13^*S* and that obtained with ordinary CO_2_ (natural abundance, no labelling) is denoted as ^12^*S*, then the ^13^C percentage is given by: %^13^C = ^13^*S*/^12^*S* × 1.1/99 × 100 where 99 and 1.1 stand for the isotopic enrichment in inlet CO_2_; (ii) using spin–spin interactions since the contribution of the side peaks to the total signal represent the %^13^C of neighboring C-atoms. The %^13^C from NMR reported here (Figure 4) is the average of these two calculations. With LC–MS, the %^13^C was calculated using the relative abundance of signals *S_k_* weighted by the number of isotopically substituted carbons (*k*): %^13^C = (Σ*k∙S*_k_)*_k_*_≥1_/*n*∙(Σ*S*_k_)*_k_*_≥0_, where *n* is the total number of carbons in the molecule. The rate of oxygenation (*v_o_*) was estimated as *v_o_* = 2v_c_C*/*c_i_* where C* is the *c_i_*-based CO_2_ compensation point in the absence of day respiration (40 µmol mol^−1^), and *v_c_* = (*A* + *R_d_*)/(1 − C*/c_i_) where *R_d_* is day respiration (here, 0.5 µmol m^−2^ s^−1^), with C* and *R_d_* obtained using the Laisk method. Note that *v_o_* is not critical at all and its impact on accumulation (build-up) rates is discussed in Section 3, Discussion. 

Calculation of build-up rates (denoted as ε, defined in Figure 2) for glycine, serine and glycerate (Figure 5) was carried out using mass-balance equations and convergence of the solution of the differential equation describing ^13^C pools. Here, we used *net* build-up rates to allow facile solving (although we recognize it may be a bidirectional flux). The input of glyoxylate by oxygenation is supposed to be fully labelled—i.e., at 100% ^13^C (we thus assumed there was no build-up and no isotopic dilution by a significant pre-existing pool). The total ^13^C-NMR signal (total observed ^13^C content, ^13^Q) integrates both metabolically active and slow turned-over pools: ^13^Q_obs_ = ^13^Q_act_ + ^13^Q_slow_. ^13^C amounts ^13^Q are described by differential equations of the general form: d^13^Q_m_/dt = ^13^C-influx − ^13^C-efflux = *i*∙p_i_ − *e∙*p_m,_ where m stands for the metabolite of interest, *i* and *e* are influx and efflux rates, and p_i_ and p_m_ are %^13^C in source metabolite i and metabolite m, respectively. In what follows, we give details for glycine. Assuming that the pool size of metabolically active glycine ($S_{Gly}^{act}$) does not vary, the elemental change in ^13^C content in active glycine with time is given by:

(2) $$\frac{d^{13}Q_{Gly}^{act}}{dt}=\frac{d\left(S_{Gly}^{act}\times p_{Gly}^{act}\right)}{dt}=S_{Gly}^{act}\times \frac{dp_{Gly}^{act}}{dt}$$

By mass-balance, it is also given by:

(3) $$\frac{d^{13}Q_{Gly}^{act}}{dt}=v_{o}\times p_{glyoxylate}-v_{o}\times p_{Gly}^{act}$$

Therefore:

(4) $$\frac{dp_{Gly}^{act}}{dt}=\frac{v_{o}}{S_{Gly}^{act}}\left(p_{glyoxylate}-p_{Gly}^{act}\right)$$

Which can be easily solved with the initial conditions (percentage *π_o_* = 1.1% (natural abundance) at *t* = 0):

(5) $$p_{Gly}^{act}\left(t\right)=p_{glyoxylate}-\left(p_{glyoxylate}-\pi_{o}\right)e^{-\;\frac{v_{o}t}{S_{Gly}^{act}}}$$

An equation similar to (2) cannot be written for the slow (metabolically “inactive”) glycine pool because its size is not constant (build-up). Thus, we have:

(6) $$\frac{d^{13}Q_{Gly}^{slow}}{dt}=\frac{d\left(S_{Gly}^{slow}p_{Gly}^{slow}\right)}{dt}=\varepsilon_{Gly}p_{Gly}^{act}$$

By integration, it gives:

(7) $$S_{Gly}^{slow}p_{Gly}^{slow}=\lambda +\int \varepsilon_{Gly}p_{Gly}^{act}dt$$

where $p_{Gly}^{act}$ is given by (5) and λ is a constant obtained from initial conditions (natural abundance) as follows:

(8) $$\lambda ={\left(S_{Gly}^{slow}\right)}_{0}\pi_{o}-\varepsilon_{Gly}S_{Gly}^{act}\frac{p_{glyoxylate}-\pi_{o}}{v_{o}}$$

When the time of observation *t* is large (here, *t* = 7200 s), the observed ^13^C amount can be approximated by:

(9) $${}^{13}Q_{Gly}^{obs}\approx p_{glyoxylate}\left(S_{Gly}^{act}+\varepsilon_{Gly}t\right)-\varepsilon_{Gly}S_{Gly}^{act}\frac{p_{glyoxylate}-\pi_{o}}{v_{o}}+{\left(S_{Gly}^{slow}\right)}_{0}\pi_{o}$$

Therefore, the build-up rate is given by:

(10) $$\varepsilon_{Gly}=\frac{{}^{13}Q_{Gly}^{obs}-\pi_{o}{\left(S_{Gly}^{slow}\right)}_{0}-S_{Gly}^{act}p_{glyoxylate}}{tp_{glyoxylate}-S_{Gly}^{act}\frac{p_{glyoxylate}-\pi_{o}}{v_{o}}}$$

Note that the right term in (10) is rather close to the quotient ^1^${}^{13}Q_{Gly}^{obs}$/*t* but corrected for the fact that observed ^13^C by NMR includes both highly ^13^C-enriched metabolically active glycine and ordinary glycine at ^13^C natural abundance. In (10), there are potentially three unknowns: *v_o_*, $S_{Gly}^{act}$, and ($S_{Gly}^{slow}$)_o_.

The oxygenation rate *v_o_* was estimated using the intercellular CO_2_ mole fraction and the *c_i_*-based compensation point in the absence of day respiration (see above). $S_{Gly}^{act}$ was estimated using the ^13^C amount seen under 2% (corrected for natural abundance) since under these conditions, there is no glycine build-up. ($S_{Gly}^{slow}$)_o_ was estimated using the observed amount in 0% O_2_ since no significant ^13^C-glycine flux occurs in this condition (negligible *v_o_*). Finally, our calculations assumed that cytoplasmic non-photorespiratory serine biosynthesis was negligible.

## 5. Conclusions

Taken as a whole, our method takes advantage of observed absolute ^13^C amounts (in moles) by NMR to provide an estimate of build-up rates of photorespiratory intermediates in illuminated leaves, using simple assumptions for calculations. Additional LC–MS routine analyses allow facile determination of isotopologue populations and are thus complementary to NMR. For a broad range of gaseous (CO_2_/O_2_) conditions, we found that the build-up rate of photorespiratory intermediates is a real phenomenon, but it is numerically very small compared to Φ and *v_o_*. While it is therefore unlikely that it causes strong alterations of N metabolism, electron transport or CO_2_ exchange during photosynthesis, metabolite build-up probably has long-term consequences for nitrogen metabolism [26]. We also recognize that we illustrated our isotopic method under optimal conditions while there could be some variations in build-up rates in response to specific environmental conditions, such as nutrient deficiency (e.g., potassium deficiency which is well-known to impact on stomatal conductance and thus *c_i_*, [27,28,29]) or water deficit [30,31]. Additionally, plants grown at high CO_2_ have an altered content in GDC-SHMT [32] and thus are more likely to have a larger glycine build-up rates under conditions promoting high photorespiration. This aspect will be examined in a future study.

## Figures and Tables

**Table 1:** Gaseous conditions used in experiments to vary the photorespiration rate ^1^.

Condition	O_2_/CO_2_	Photorespiration
1	100/380	Very high
2	21/140	High
3	21/380	Normal
4	21/800	Low
5	2/380	Very low
6	0/380	Negligible

^1^ O_2_/CO_2_ conditions are given in %/µmol mol^−1^. O_2_ is indicated in % of background gas (balanced with N_2_).

## Data Availability

Source data are available on request to the corresponding author.
